# *Porphyromonas gingivalis* and adverse pregnancy outcome

**DOI:** 10.1080/20002297.2017.1374153

**Published:** 2017-09-13

**Authors:** Leticia Reyes, Priscilla Phillips, Bryce Wolfe, Thaddeus G. Golos, Molly Walkenhorst, Ann Progulske-Fox, Mary Brown

**Affiliations:** ^a^ Department of Pathobiological Sciences, School of Veterinary Medicine, University of Wisconsin - Madison, Madison, WI, USA; ^b^ Microbiology & Immunology, A.T. Still University of Health Sciences, Kirksville College of Osteopathic Medicine, Kirksville, MO, USA; ^c^ Wisconsin National Primate Research Center, University of Wisconsin – Madison, Madison, WI, USA; ^d^ Department of Comparative Biosciences, School of Veterinary Medicine, University of Wisconsin – Madison, Madison, WI, USA; ^e^ Department of Oral Microbiology, Center for Molecular Microbiology, University of Florida, Gainesville, FL, USA; ^f^ Infectious Disease and Immunology, College of Veterinary Medicine and D. H. Barron Reproductive and Perinatal Biology Research Program, University of Florida, Gainesville, FL, USA

**Keywords:** *Porphyromonas gingivalis*, preeclampsia, preterm delivery, fetal growth restriction, defective deep placentation, uterine NK cells, decidual macrophages

## Abstract

*Porphyromonas gingivalis* is a Gram-negative, anaerobic bacterium considered to be an important pathogen of periodontal disease that is also implicated in adverse pregnancy outcome (APO). Until recently, our understanding of the role of *P. gingivalis* in APO has been limited and sometimes contradictory. The purpose of this review is to provide an overview of past and current research on *P. gingivalis* that addresses some of the controversies concerning the role of this organism in the pathogenesis of APO.

## Introduction

*Porphyromonas gingivalis* is a Gram-negative, anaerobic bacterium considered to be an important pathogen of periodontal disease [1]. As a periodontal pathogen, *P. gingivalis* may indirectly contribute to adverse pregnancy outcomes (APO) by facilitating the release of bacterial products or inflammatory mediators into the maternal circulation that reach the maternal-fetal interface [2]. *P. gingivalis* could also directly promote APO via invasion and injury to utero-placental tissues; this is supported by several studies that have detected *P. gingivalis* DNA/antigen in the placenta, amniotic fluid, umbilical cord, and neonatal nasogastric aspirates from complicated pregnancies [3–11]. However, the significance of *P. gingivalis* as a causative agent of APO is sometimes viewed with skepticism due to several confounding factors. For instance, periodontal disease in of itself is a poor predictor of preterm birth, preeclampsia, fetal growth restriction, or perinatal death [2,12]. Moreover, a consensus of interventional studies in which pregnant women received periodontal treatment did not show an overall significant reduction in the rate of preterm birth, fetal growth restriction, low birth weight, stillbirth, or miscarriage in these cohorts [2,12]. Also confounding is that *P. gingivalis* DNA or antigen has also been detected in placentas from healthy pregnancies, albeit at a lower microbial load and lower frequency than women with preeclampsia or preterm birth [3–7]. It is also difficult to extrapolate the contribution *P. gingivalis* to APO when its detection in utero-placental tissues is usually in association with other oral bacterial species [3–5], or when it is not detected in placental tissues from complicated pregnancies even when other oral bacterial species are present [13].

Experimental infection in various animal models confirms that invasion of the uterine compartment by *P. gingivalis* produces a diverse array of APO, including utero-placental pathology, enhanced expression of pro-T helper (TH)1 type cytokines (IFN-γ, IL-2, IL-12, and TNF-α), fetal growth restriction (FGR), and spontaneous preterm delivery [12–15]. Since these outcomes are manifestations of different pathologic mechanisms within the fetal compartment, it may seem somewhat perplexing that one organism could be responsible for all of these complications. For example, FGR can be a sequela of abnormal placentation or placental angiogenesis, and can accompany severe or early onset preeclampsia [14]. Inflammation associated with FGR or preeclampsia is usually maternal in origin [14]. On the other hand, preterm labor is a complication of overt inflammation that begins in the fetal sac and is usually initiated by activation of toll-like receptors or inflammasomes [15,16]. In this scenario, low birth weight is a result of prematurity rather than placental dysfunction that restricts proper growth of the fetus.

Recent studies regarding the pathogenic mechanisms of *P. gingivalis* provide new insights into the role of this organism in periodontal disease and/or APO. The purpose of this review is to provide an overview of past and current research on *P. gingivalis* that addresses some of the controversies concerning the role of this organism in the pathogenesis of APO. In addition to presenting new data generated by our research group, we searched PubMed for publications relevant to the topic of this review. Search terms for this review included: *Porphyromonas gingivalis*, periodontal disease, periodontitis, gingivitis, hormones and pregnancy, preeclampsia, preterm delivery, fetal growth restriction, defective deep placentation, uterine NK cells, and decidual macrophages.

## The interaction between pregnancy-related hormones and oral health

The phenomenon of pregnancy-induced gingivitis is widely reported [17,18], and our current understanding of the link between oral disease and APO indicate that any associated risk is likely precipitated by early or preexisting oral disease. While hormonal changes have been shown to increase bleeding on probing and gingival inflammation in healthy women with normal plaque index and a healthy periodontium, this has been demonstrated to occur independently of pro-inflammatory cytokines IL-1β and TNF-α, which are involved in recruitment of neutrophils and monocytes [17]. These findings indicate that there is direct hormonal induction of gingivitis that does not involve a host response to oral plaque. This finding is significant in that it demonstrates that a clinical diagnosis of gingivitis during pregnancy is not necessarily indicative of oral disease. In a diseased state, pregnancy has been shown to exacerbate existing oral disease, even in the absence of a notable increase in oral plaque [19]. In normal uncomplicated pregnancies, susceptibility to pregnancy-induced gingivitis is reported to occur between 12 and 28 weeks of pregnancy [20]. These findings support the conclusion that should periodontal intervention reduce APO for individuals at risk, the intervention should be performed as soon as a woman knows she is pregnant or (preferably) before becoming pregnant.

In general, sex hormones are reported to increase vascular permeability and proliferation while inhibiting oral mucosal tissue repair, suggesting that sex hormone associated gingivitis (e.g. puberty, menstruation, pregnancy) alters the effectiveness of the epithelial barrier to the oral microbiota [17,18,21]. One study demonstrated that both *Prevotella intermedia* and *P. gingivalis* are able to reduce testosterone to 5-alpha dihydrotestosterone (DHT) and induce DHT synthesis by fibroblasts [22]. More recently, a study investigating the role of androgen regulation during placentation showed that the placenta uses both androgen receptors and histone lysine demethylases to mediate androgen signaling and epigenetic regulation of gene expression during placental development [23]. These authors suggested that abnormal androgen signaling might alter placental development. Notably, it has been demonstrated through multiple animal and human studies that androgens play a role in APOs [24,25]. In such a scenario, one may imagine that early placental colonization by microorganisms like *P. gingivalis* that are capable of interfering with androgen signaling may adversely impact placental development through this mechanism.

Female sex hormones may directly promote microbial growth, as they stimulate *P. intermedia* growth [26,27] and an increase in both *P. intermedia* and *P. gingivalis* in response to hormonal changes during pregnancy [17,19] has been reported. However, due to variable and sometimes conflicting reports in the literature, there is no strong consensus linking increased concentrations of female sex hormones and an increased number (microbial load) of specific periodontal pathogens. Rather, such studies highlight the complexity of the interactive relationships between bacterial constituents of the oral microbiota. Moreover, it has been suggested that sex hormones participate in signaling between pathogens and hosts; therefore, studies that reported sex-linked association of susceptibility to infectious disease may involve microbial responses (e.g. expression of virulence factors) other than just microbial proliferation [28]. Thus, it has been difficult to develop better criteria for identifying ‘at risk’ individuals, since it is a challenge to define the underlying mechanisms responsible for the reported associations between APO and periodontal disease, APO and certain oral community profiles, and/or APO and specific oral pathogens.

## Periodontal disease, TH17/Treg cell imbalance, and APO

TH17 cells and IL-17 are reported to increase in periodontal lesions and exacerbate the *P. gingivalis* ligand-induced inflammatory process [29]. More recently, it has been demonstrated that *P. gingivalis* favors T helper cell polarization to a TH17 profile with generation of TH17-related cytokines [30] and that the *P. gingivalis* induced T cell differentiation shift is highly specific towards a TH17 cell phenotype in an IL-6 dependent manner [31]. Moreover, a periodontitis rat model study showed a variable site-specific TH17/Treg cell ratio in the oral tissues but detected a TH17 cell increase with a relative Treg cell decrease in peripheral blood, which was proposed to potentially impact development of systemic inflammatory diseases [32]. This hypothesis is supported by a recent study that found that *P. gingivalis-*induced periodontitis exacerbates arthritis in an IL-17RA (receptor A) dependent manner in an antigen-induced arthritis murine model [33]. The balance of TH17 cells relative to Treg cells is a central component to balancing the immune response such that pathogens are appropriately recognized, but inappropriate immune responses to self and harmless antigens are minimized [19]. Pregnancy requires recalibration of this balance to protect the mother from infection while accepting a semi-allogenic fetus. Multiple studies have shown that an excess of TH17 with a reduced Treg cell profile can lead to APO and is reviewed in detail by Figueiredo and Schumacher, 2016 [34]. Therefore, it is reasonable to hypothesize that a combination of preexisting periodontal disease and *P. gingivalis* placental colonization would increase the risk of APO in part by inducing a TH17/Treg cell imbalance.

## Could *P. gingivalis* affect the placental microbiome?

**Figure 1.**
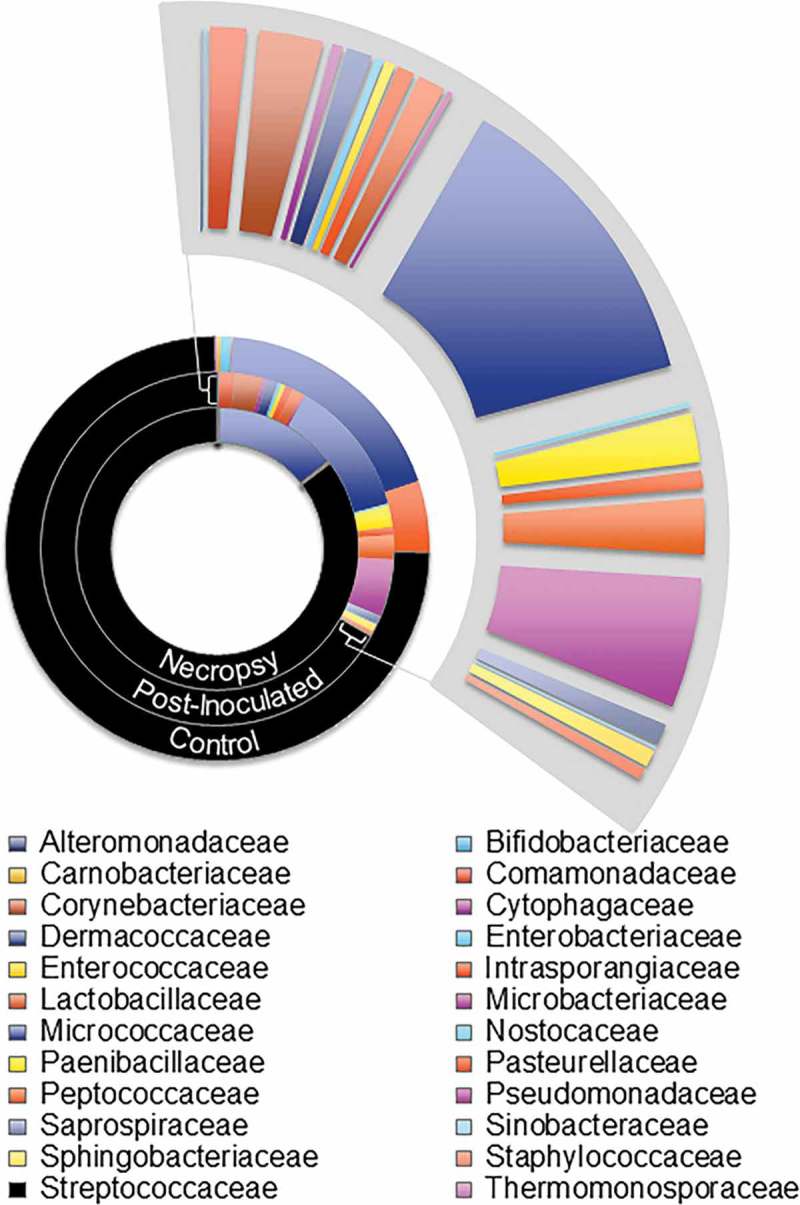
Consensus oral microbiome profiles of Sprague-Dawley (SD) female rats at the family taxonomic level. The consensus data for pre-inoculation baseline, post-P. gingivalis inoculated before breeding, and pregnant (gestation day 18) necropsy were used to generate this family-level comparison (results have not been published elsewhere). Female rats received repeated oral inoculations with *P. gingivalis* strain A7A1-28 as previously described [43]. Oral samples were collected 7 days after the last inoculation (post-*P. gingivalis* group). Breeding was started 2 weeks after the last oral inoculum and dams were sacrificed at gestation day 18.

The paradigm that the placenta is a sterile environment has been challenged by several reports that show bacterial DNA or live bacteria present in placentas obtained from normal pregnancies [3–5,35–38]. Interestingly, one study that used comparative 16s ribosomal DNA-based and whole-genome shotgun metagenomic analyses on 320 placental specimens reported that the placental microbiome most closely resembles supragingival plaque [35]. Subsequent studies do not necessarily describe the same microbial community composition within the placenta [36,39,40]. However, they do report a link between APO and placental dysbiosis, such as enrichment of *Fusobacterium* species in placentas from preterm and low-birth-weight pregnancies [36,40] or trace levels of *Porphyromonas*, *Prevotella*, *Variovorax*, and *Dialister* in placentas from preeclamptic women [39]. Given that *P. gingivalis* promotes oral dysbiosis [41,42], we propose that this may be an important mechanism through which *P. gingivalis* contributes to adverse pregnancy outcomes. In a pilot study conducted by our research group, we examined the composition of the oral microbiome of Sprague-Dawley (SD) rats that received repeated oral inoculations of sterile vehicle or 10^9^ CFU of *P. gingivalis* strain A7A1-28 (Figure 1). Oral swabs were collected before the inoculation procedure was started, at the end of the 3-month inoculation phase, and at time of necropsy when animals were at gestation day 18. Metagenomic sequencing targeting seven of the nine hypervariable regions (V2, V3, V4, V6–7, V8, and V9) of the 16S bacterial rRNA gene was performed using the Ion 16S Metagenomics Kit (Thermo Fisher Scientific, Waltham, MA, USA). Our approach showed that Streptococcaceae and Micrococcaceae families collectively comprised about 80–95% of the oral microbiota of SD rats and consistently dominated the oral microbiota at each time point (Figure 1). Consistent with previous reports [41], we found that infection with *P. gingivalis* strain A7A1-28 induced oral dysbiosis that persisted during pregnancy, albeit with a different composition than before pregnancy. While a relatively minor but notable proportional population decrease for both Streptococcaceae and Micrococcaceae families was observed at the end of the 3-month A7A1-28 inoculation phase, this observation coincided with an increase in population proportion of other microbial families and, more importantly, a dramatic increase in diversity in the oral microbiome. While a downward shift in oral microbial diversity was observed at necropsy of the A7A1-28 infected and pregnant SD rats, they maintained a greater oral microbial diversity relative to control. Interestingly, the Pasteurellaceae family, which constituted less than 1% of the total population in both the control and 3-month A7A1-28 post-inoculation microbiota, shifted to a consensus population proportion of 5.47% at necropsy. This family was identified to primarily consist of *Haemophilus parainfluenzae –* an opportunistic pathogen associated with a wide range of infections, including intra-abdominal and genital infections, and a common colonizer of the mucosa [44]. Although the literature to date is relatively sparse, *H. parainfluenzae* is also a species of concern with respect to mother-to-infant infections and APO [44–47]. Our findings further support the hypothesis that *P. gingivalis-*induced dysbiosis results in an oral microbiome profile that permits colonization of potential pathogens and favors an increase in population proportion of resident opportunistic pathogens, increasing the overall risk of microbial infection-associated APOs.

Hajishengallis et al. [41] demonstrated that *P. gingivalis* disrupts the composition of the oral community structure, facilitating the overgrowth of oral commensals that in turn enhance oral inflammation. A significant feature of this study is that it showed that the commensal oral microbiota must be present in order for *P. gingivalis* to induce periodontal disease. Nakajima et al. [42] have recently shown that *P. gingivalis*-mediated dysbiosis is not limited to the oral cavity. Specifically, oral inoculation of C57BL/6 mice with *P. gingivalis* increases the proportion of *Bacteroidetes* and decreases the proportion of *Firmicutes* within the ileum, with subsequent increased gut permeability and dissemination of gut bacteria into the liver. To date, it has not been determined whether *P. gingivalis* enhances intrauterine infections by other microbes, but there is a rationale for conducting such experiments. Monotypic infection with *P. gingivalis* in pregnant rodents produces lesions that disrupt the maternal-fetal barrier involving inflammation of uterine arteries and infiltration of the uterine submucosa by neutrophils [48]. These lesions could facilitate invasion of the placenta by other bacteria that gain entry into the uterus, since polymicrobial colonization/infections are more common at the maternal-fetal interface than monotypic infections [3–5,8,9,39].

The microbial community-wide dysbiotic effect of *P. gingivalis* is largely driven by this microbe’s ability to subvert host antimicrobial defenses without suppressing inflammation, which in turn facilitates the growth of bacterial species that can tolerate and exploit the inflammatory environment (reviewed by Zenobia and Hajishengallis [49]. Although the polymicrobial etiology of inflammatory diseases such as periodontal disease is associated with dysbiosis of the commensal microbiota, disease susceptibility may depend on an individual’s tolerance to dysbiosis in the context of intrinsic host factors such as genotype and immunological status, or extrinsic host factors such as diet, stress, and behavior. This is further predicated on the relative pathogenicity (e.g. virulence factors) of the constituent members of the microbiota. While there are several *P. gingivalis* virulence mechanisms that promote dysbiosis [49], the ability of *P. gingivalis* to synthesize different lipopolysaccharide (LPS) structures that either activate or antagonize TLR4, such as lipid A1 and 4′ phosphatases [50], is particularly relevant to the maternal-fetal interface. The uterus, placenta, and fetal membranes express TLRs 1–9 and dysregulation of TLR function in these tissues can promote APO [51,52]. In the oral cavity, modification of *P. gingivalis* lipid A is required for successful colonization and overgrowth of plaque bacteria [50]. This was confirmed *in vivo* with a ligature model of periodontitis in rabbits that were inoculated with *P. gingivalis* strain A7436 or lipid phosphatase mutants that were ‘locked’ into producing a TLR4 agonist or antagonist [50]. In this study, all A7436 lipid A mutants altered the composition of the oral microbiota, but only TLR4 antagonist and wild-type A7436 enhanced the growth of *Fusobacterium nucleatum*, which is implicated in preterm delivery [53,54] and it is often found in association with *P. gingivalis* in placenta, amniotic fluid, and nasogastric aspirates from preterm infants [4,8]. Furthermore, the placenta is a blood-rich tissue and red blood cells contain hemin. At least *in vitro*, hemin-rich medium promotes the growth of TLR4 antagonist forms of *P. gingivalis* [55], and thus the placental environment may be enriching for this lipid A structure. This notion is implied by results from a previous study that examined the impact of *Campylobacter rectus* and *P. gingivalis* strain A7436 coinfection in a rodent model of APO [56]. Specifically, Arce et al. found that coinfection with A7436 reduced *C. rectus*-induced expression of TLR4 in the placenta, but produced FGR that did not occur in animals singly infected with *C. rectus* [56]. *P. gingivalis*-induced FGR is indeed associated with an increased placental expression of TH1-type cytokines: IFN-γ, IL-2, and IL-12 with a concurrent reduction in TH2 type IL-10 and TGF-β2 [57]. It should be noted that FGR is a syndrome with multiple pathologic etiologies [14,58]. Underlying causes of FGR include inflammation from unknown causes or secondary to hematogenous infection, as well as underlying vasculopathies such as poorly remodeled uterine arteries or abnormal placental angiogenesis [58].

## P. gingivalis as a pathogen of the placental bed

### Uterine vascular changes during pregnancy

Although a thorough understanding of uterine changes during pregnancy is beyond the scope of this review, it is important to summarize some key features of this process in order to appreciate the impact *P. gingivalis* has on this system. Successful pregnancy depends on adequate remodeling of uterine spiral arteries from narrow lumen/high resistance vessels to dilated/low resistance vessels that lack their musculoelastic wall. Optimal transformation of the uterine arteries allows the placenta to receive an adequate blood supply without pulsatile turbulent flow that could damage the placenta. The actual process of spiral artery remodeling involves endothelial cell swelling, separation and dedifferentiation of vascular smooth muscle cells (VSMC), degradation of the extracellular matrix with deposition of fibrinoid material, and infiltration of the vessel wall with invading trophoblasts [59]. Effective spiral artery remodeling depends on a series of coordinated events between arterial endothelium, VSMC, uterine natural killer cells (uNK), macrophages (MΦ), and extravillous trophoblasts (EVT) [59]. Changes in endothelial cells and VSMC begin early during decidualization, which is driven by increasing concentrations of progesterone and estrogen [60].

During early pregnancy, uNK cells make up ~70% of leukocytes within the placental bed [61,62]. uNK cells have a distinctly different phenotype than circulating NK cells in that they are CD56^bright^/CD16^−^ and express a repertoire of activating and inhibitory receptors (NKRs) that can fluctuate in response to EVT and invading pathogens [63]. Although the biological functions of uNK cells is still largely unknown, these cells appear to be important for spiral artery remodeling. Experimental ablation of uNK cells in pregnant rats reduces EVT invasion and induces vascular and perivascular necrosis with fibrosis surrounding the artery [64]. Human uNK cells induce disruption of VSMC and breakdown of the extracellular matrix *in vitro*, possibly making the arteries more penetrable to EVT cells [65]. Their role in vascular remodeling is also implied by their phenotype. UNK cells produce angiogenic factors (VEGF, placental growth factor, and angiopoietin 2), cytokines (GM-CSF, IFN-γ, CSF-1, and TNF-α), matrix metalloproteinases, and surface receptors (NK complex of lectin related genes and leukocyte receptor complex of immunoglobulin-related genes) that interact with MHC class I molecules uniquely expressed by EVT (HLA-G, HLA-E, and HLA-C) [62].

Macrophages comprise ~20% of the decidual leukocyte population [61]. Unlike uNK cells, which predominate during the first trimester of pregnancy, macrophages maintain their levels within the decidua throughout pregnancy [61]. However, macrophages perform similar tasks in that they also regulate the remodeling of the spiral arteries through release of various cytokines, matrix metalloproteinases, and angiogenic factors [66]. Decidual macrophage (dMΦ) populations are heterogeneous, but most tend towards M2 polarity phenotypes (M2a, M2b, and M2c) based on their expression of IL-10, TGF-β, indoleamine 2,3-dioxygenase (IDO), IL-6, and/or TNF-α [67], and their ability to suppress uNK cytotoxicity toward EVT [68]. dMΦ can minimize inflammation by phagocytosing cellular debris from apoptotic VSMC [67], but also have the capacity to revert to a pro-inflammatory phenotype, as seen in recurrent miscarriage, preeclampsia, and fetal growth restriction [67,69].

EVTs facilitate spiral artery remodeling by promoting apoptosis of arterial endothelium and VSMC [70]. Another important feature of EVTs is that they express unique MHC class I molecules (HLA-G, E, and C), which maintain maternal tolerance of the fetus via immune modulation of uNK cells, macrophages, and T cells [71]. Thus, anything that blocks EVT invasion into the uterus or inappropriately leads to EVT death could result in implantation failure or poor placentation.

It is important to note that EVTs are functionally distinct from villous trophoblasts. First, EVT are highly motile and capable of invading the uterus, whereas villous trophoblasts are not [70]. Second, EVTs are susceptible to invasion by intracellular bacteria [72], unlike villous trophoblasts, which are highly resistant to microbial invasion [73]. During the first trimester of pregnancy when EVTs form anchoring villi to attach the placenta to the decidua and the uterine wall, this susceptibility affords the opportunity for vertical transmission of pathogens to the fetus [72].

### The impact of impaired spiral artery remodeling on pregnancy

Inadequate remodeling of the myometrial segments of the uterine spiral arteries is referred to as defective deep placentation (DDP) [74,75]. Studies of DDP are challenging because adequate specimens can only be obtained by invasive biopsy, hysterectomy, or from cadavers [75]. Despite this limitation, it has become clear that DDP underpins a spectrum of obstetric complications ranging from first- and second-trimester spontaneous abortion, preterm premature rupture of membranes (PPROM), and spontaneous preterm birth to intrauterine growth restriction (IUGR) and abruption placentae [75,76]. These complications are often referred to as the great obstetrical syndromes (GOS) [75]. Remarkably, each of these obstetric disorders has different clinicopathologic features within the placenta and/or fetal membranes that set them apart from each other [75,76]. The diversity of the GOS underscores the significance of DDP as a central pathologic mechanism that begins during the earliest weeks of pregnancy before any complications manifest. Thus, any effective strategy to prevent DDP would require early intervention, possibly even before pregnancy begins.

### *P. gingivalis* as a vascular pathogen of the placental bed

Given the difficulty in obtaining human placental bed specimens, it is not surprising that there is no information to identify whether *P. gingivalis* colonizes the deep uterine vasculature or if this is linked to APO. However, there are some implications that support this notion. The inner third of the placental bed, the decidua, ‘peels off’ with the placenta when it is removed, so it is readily available for analysis. Studies that have sampled the decidua for the presence of *P. gingivalis* DNA reported that 70–92% of specimens from women with preeclampsia were positive [4,5]. Another study that screened a very preterm cohort (≤32 weeks’ gestation) consisting of spontaneous preterm birth, small for gestational age, preeclampsia, and preeclampsia with HELLP syndrome (hemolysis, elevated liver enzymes, and low platelet count) cases found the presence of *P. gingivalis* within the placenta significantly linked to shorter gestation lengths (OR 0.63 [95% CI: 0.48–0.85]; p = 0.002), regardless of the diagnosis of APO [6]. Although spontaneous preterm birth, small for gestational age, preeclampsia, and preeclampsia with HELLP syndrome have different pathologies within the placenta, DDP can be found in all these disorders [75]. Thus, DDP could be a common mechanism by which *P. gingivalis* could contribute to APO.

**Figure 2.**
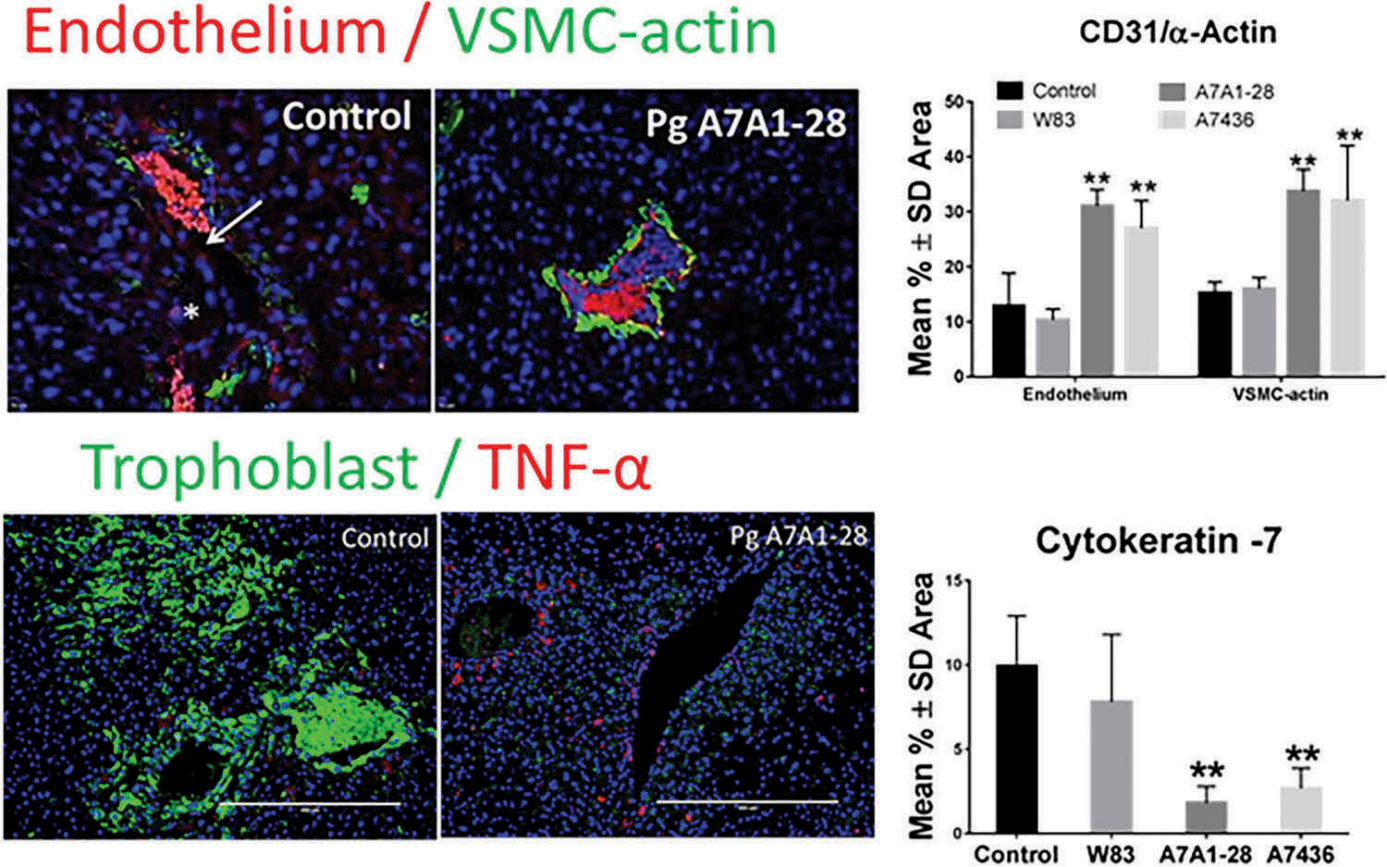
Infection with P. gingivalis strains A7A1-28 and A7436 but not W83 impairs remodeling of uterine spiral arteries in SD rats. Animals received repeated oral inoculations of sterile vehicle (control) or *P. gingivalis* as described in Figure 1. Endothelial cells were detected with rabbit polyclonal antibody to CD31 (Abbiotec™, San Diego, CA). VSMC were identified with anti-α-actin mouse clone 1A4 (Biorad Laboratories, Hercules, CA). EVT were detected with anti-cytokeratin 7 mouse monoclonal antibody clone LP1K (Abcam®, Cambridge, MA) and nuclei were stained with DAPI. Calibrated digital images were analyzed with Image J 1.50b analysis software (Rasband; National Institutes of Health, USA). Morphometry of spiral artery remodeling was performed with the particle analysis feature. Data were analyzed by one-way ANOVA and Tukey’s tests. **Values different from control and W83 p < 0.01 (n = 5). Unpublished data were presented at the International Federation of Placenta Associations meeting held in Portland, OR, September 2016.

**Figure 3.**
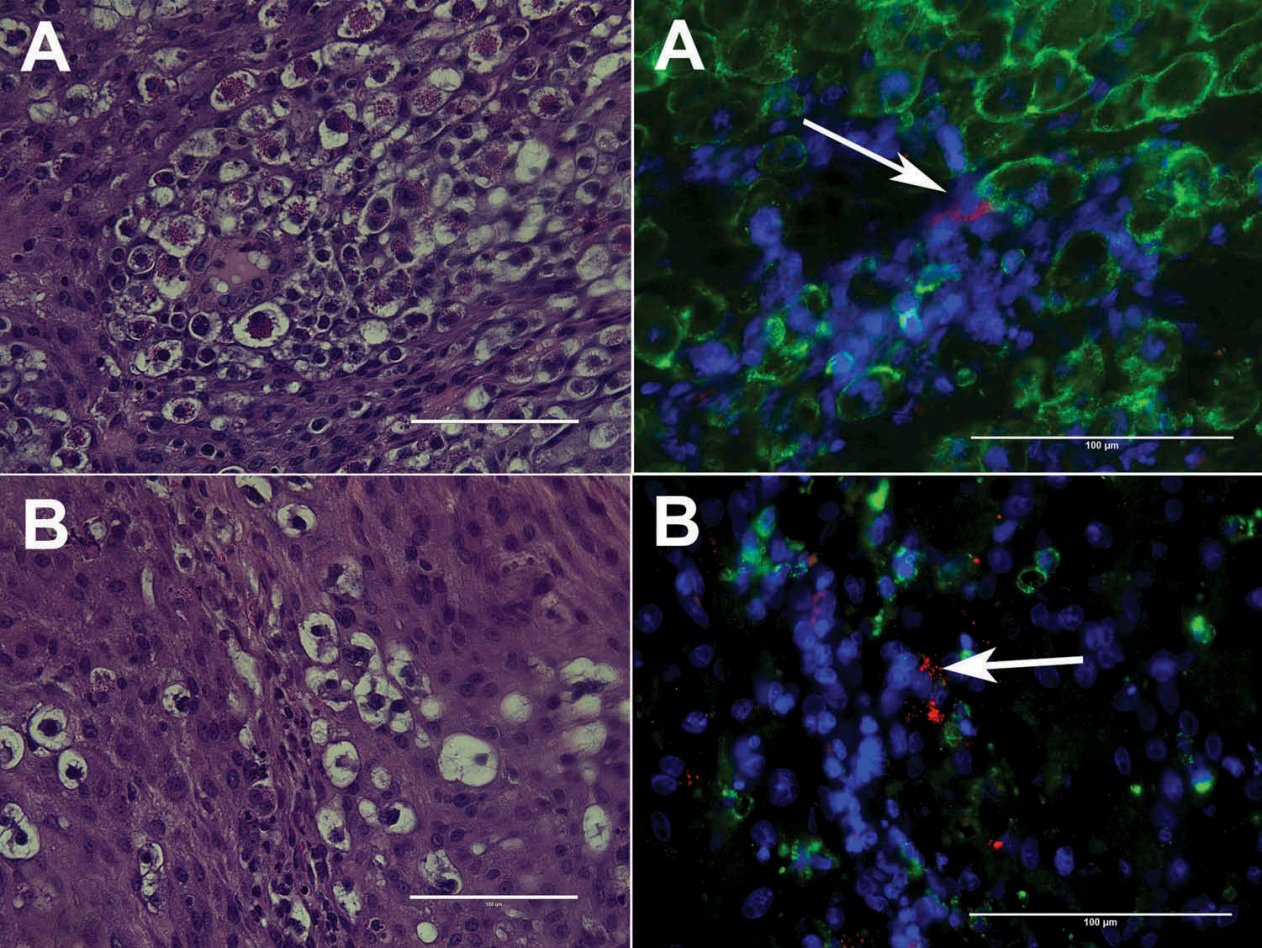
Representative images of *P. gingivalis* antigen in the metrial triangle of SD rats in association with uNK cells (A) or CD68^+^ decidual macrophages (B). P. gingivalis (red) was detected with a rabbit polyclonal antibody to whole cell W83 [6,77], uNK cells were identified with mouse monoclonal antibody clone ANK61 to NK cell activation structure, decidual macrophages were labeled with anti-CD68 mouse clone ED1antibody (Abcam®, Cambridge, MA), and nuclei (blue) were stained with DAPI (results have not been published elsewhere).

Both *in vivo* and *in vitro* infection models indicate that *P. gingivalis* can interfere with remodeling of the uterine spiral arteries. In a pregnant rat model of infection, Belanger et al. were the first to provide evidence that *P. gingivalis* induces metrial arteritis [48]. This lesion was most common in dams inoculated with *P. gingivalis* strain A7436. Subsequent studies by our research group have demonstrated that infection with fimbriae-expressing *P. gingivalis* strain A7436 or A7A1-28 does indeed interfere with the physiologic remodeling of the uterine spiral artery in rats (Figure 2). In this model, reduced spiral artery remodeling is characterized by retention of arterial endothelium and VSMCs, with a significant reduction in EVT density in the placental bed. In these tissues, *P. gingivalis* antigen is most commonly detected in stromal cells that are often associated with uNK cells and CD68^+^ macrophages (Figure 3), opening the possibility that *P. gingivalis* may be perturbing the function of these cells in a paracrine manner.

**Figure 4.**
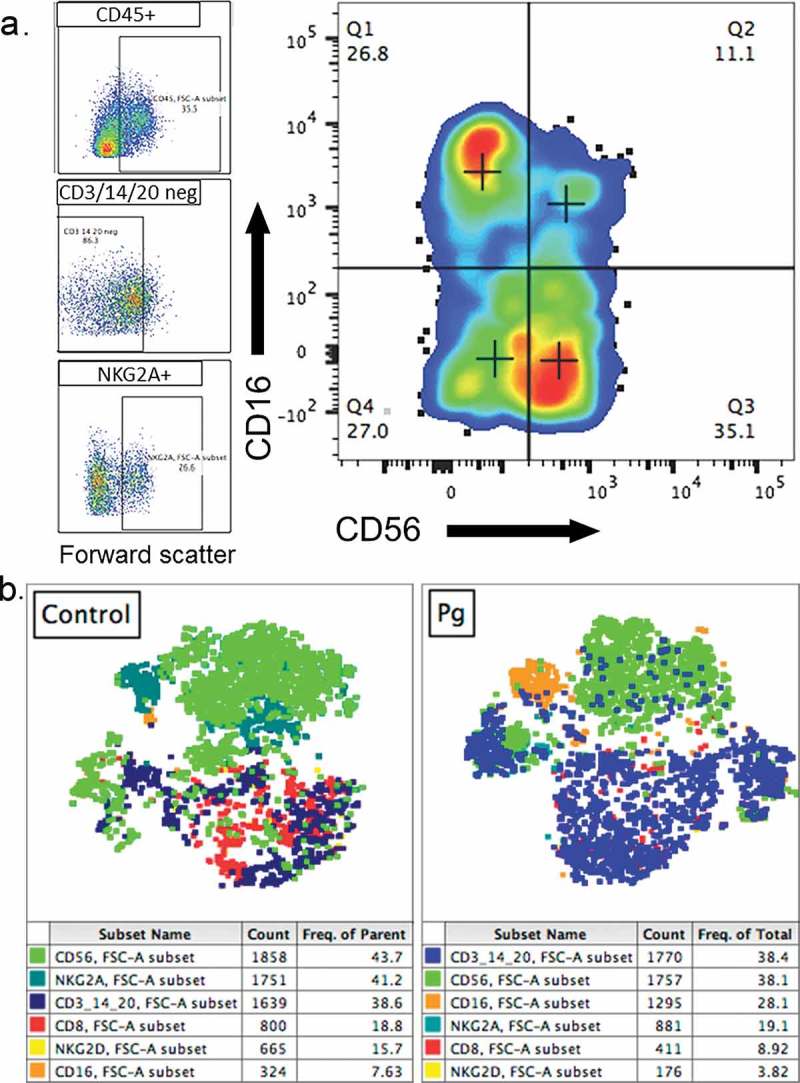
Representative manual gating strategy performed with FlowJo v10.3 software to identify dNKs defined as live, single cells that were CD45^+^/CD14^–^/CD20^–^/CD3^–^/NKG2A^+^ (A). Representative unbiased, multidimensional tSNE clustering analysis of first trimester decidual leukocytes conducted with Bioconductor package Cytofkit (B). tSNE clustering analysis revealed a greater proportion of CD3^+^/CD14^+^/CD20^+^ cells and lower proportion of NKG2A^+^ and CD56^+^ cells with *P. gingivalis* (Pg) infection compared with controls (bottom row). Each dot represents an individual cell, colored by marker expression as shown in the corresponding table below each graph (results have not been published elsewhere).

A preliminary study conducted by our group in nonhuman primates suggests that infection with *P. gingivalis* alters decidual NK cell populations *in vivo*. Pregnant rhesus macaques received  a total of three intravenous inoculations of sterile vehicle or 105 CFU of *P. gingivalis* strain A7A1-28 that were administered a week apart during the first trimester of pregnancy. Animals were euthanized at gestation day 50 ± 3 days (latter part of the first trimester; term = gestation day 165). Decidual cells were isolated as previously described [78] and evaluated by flow cytometry (Figure 4). Data analysis was performed manually with FlowJo v10.3 (Flow Jo, LLC, Ashland, OR) and also with Bioconductor package Cytofkit [79] to generate unbiased, multidimensional t-distributed stochastic neighbor embedding (tSNE) [80] population cluster maps. With a panel designed to define NK cell subsets in the decidua (dNKs), we observed a decrease in the absolute number, percentage, and median fluorescence intensity of CD56^bright^ dNK cells during *P. gingivalis* infection, with a concomitant increase in CD16^+^ NK cells. Both of these subsets had increased expression of cytotoxicity receptor NKp46 and activation marker NKG2D with infection; however, we did not observe significant differences in the expression of activation marker CD69, proliferation marker Ki-67, or inhibitory marker PD-1 in dNKs from our infected versus control cohorts.

While CD16^+^ NK cells are the predominant NK cell type in peripheral blood, they have high cytotoxicity and are present at much lower levels in the decidua. In a healthy pregnancy, most dNKs are CD56^bright^ cells that possess granules such as perforin and granzyme B but have low potential for degranulation and are mainly pro-angiogenic, cytokine secreting and immunomodulatory. Double-negative or CD56^dim^ populations have been described as secreting low levels of IFN-γ and TNF-α and having a detrimental impact on pregnancy [63]. A number of studies have found that alterations in dNK subsets are higher in cases of unexplained recurrent miscarriage, infertility, and implantation failure [81–84]. In particular, Giuliani et al. [82] found that maintaining a low ratio of CD16^+^ to CD56^+^ cells appears to be important for pregnancy success, likely due to the pro-inflammatory nature of CD16^+^ cells.

Our preliminary data suggest that infection with *P. gingivalis* induces loss of CD56^+^ dNK cells and an increase in CD16^+^ dNK cells in the first trimester. The ratio of CD16^+^ cells to CD56^+^ cells increases to 0.63 with infection versus 0.32 in control animals, and to a lesser extent so does the ratio of NKp46^+^ cells to CD56^+^ cells with infection (0.24) versus control (0.20), indicating a shift towards an activated phenotype [85,86]. There is evidence that CD56^bright^ cells have a crucial role in implantation, trophoblast migration, and spiral artery remodeling [64], and that these cells closely associate with dMΦ and decidual vessels in the first trimester [87,88].

Although our flow cytometric analysis of macaque decidual cells was not designed to comprehensively investigate T cell lineage and function (Figure 4), we did observe a higher percent and absolute count of CD3^+^ T cells in infected animals (data not shown). Within this subset, *P. gingivalis* infection also correlated with higher expression of PD-1 (46.8 ± 7% vs 4.35% control) and lower expression of IFN-γ (3.3 ± 1.5% vs 41% control), which may contribute to chronic infection by suppressing adaptive T cell responses.

**Figure 5.**
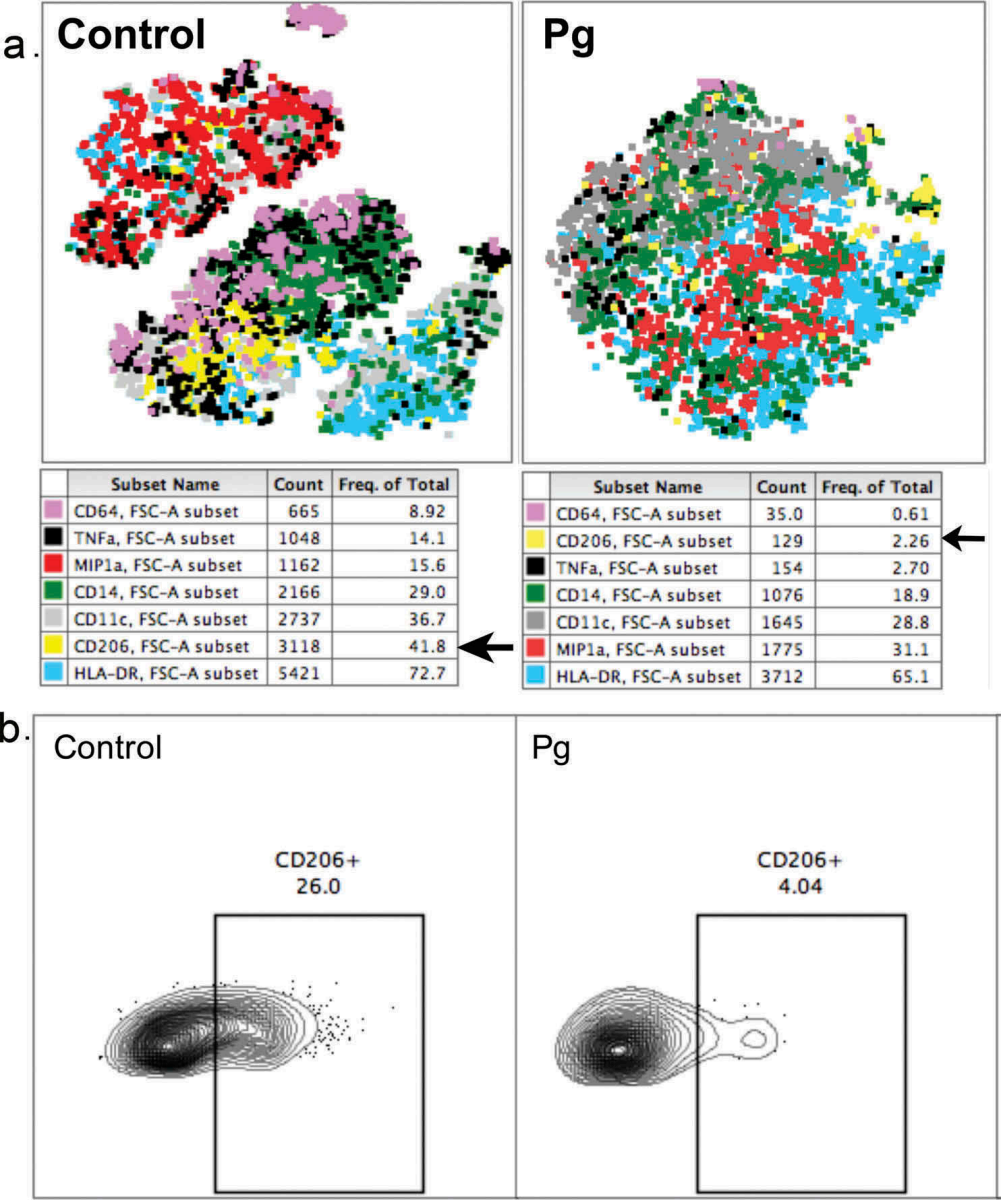
Unbiased, multidimensional tSNE clustering analysis of first-trimester decidual macrophages from uninfected and *P. gingivalis* infected macaques (A) shows a greater proportion of MIP1a^+^ cells and a lower proportion of CD206^+^ cells with *P. gingivalis* infection compared with controls. Each dot represents an individual cell, colored by marker expression as shown in the corresponding table below each graph. (B) Manual gating with FlowJo v10.3 confirms a decrease in CD206^+^ macrophages. Value indicates percentage of CD45^+^ cells. Pg = infected with *P. gingivalis*; Control = uninfected, gestational age-matched control.

In addition to lymphocytes, we examined markers of macrophages in the decidua to assess whether *P. gingivalis* infection alters the phenotype or function of these cells. As already mentioned, dMΦ also appear to have a role in spiral artery remodeling and trophoblast migration, typically bear markers associated with tissue remodeling and phagocytosis of debris, and have recently been divided into two subsets: one more regulatory, and the other more pro-inflammatory [67]. *P. gingivalis* infection resulted in a notable decrease in CD206^+^ dMΦs (Figure 5). CD206 is a marker of alternative activation and a mediator of phagocytosis of pathogens and debris, with a role in tissue remodeling [89]. A greater proportion of control dMΦs expressed CD206 in our pilot study compared with infected dMΦs (40% vs 3 ± 0.6%). A loss of alternatively activated macrophages in early pregnancy could have detrimental consequences on spiral artery remodeling [88]. Interestingly, although *P. gingivalis* infection is associated with chronic inflammation, dMΦs from infected animals appeared to secrete less TNF-α than dMΦs from controls. We observed only small decreases in FcγR1 receptor, CD64 and glycoprotein, CD1c with *P. gingivalis* infection, and no significant difference in HLA-DR (MHC class II) or proliferation marker Ki-67 expression between infected and control dMΦs. Although we cannot rule out the possibility that some of these differences are due to the variation inherent to a genetically diverse nonhuman primate model rather than a disease state, these results provide a possible mechanism for *P. gingivalis*-mediated effects on spiral artery remodeling. Further characterization will be necessary to determine the biological significance of our findings.

EVTs may also be a target for *P. gingivalis*-mediated DDP. HTR8/SvNeo cells are human EVTs [90,91] that were originally isolated from first trimester placental tissue and immortalized with the pSV3neo plasmid [92]. HTR8/SvNeo cells retain the EVT phenotype (i.e. the capacity to invade and secrete degradative enzymes) of the parent cell [92] and have been used extensively to elucidate *P. gingivalis* effects on trophoblasts *in vitro. P. gingivalis* can invade HTR8/SvNeo cells and inhibit their proliferation through induction of G1 arrest and apoptosis [93,94]. Further characterization of this phenotype shows that invasion of HTR8/SvNeo by *P. gingivalis* induces IFN-γ expression [94] and activates the DNA damage response in these cells that is facilitated by gingipain degradation of the p53 antagonist, MDM2 [95]. While EVT death could explain decreased density of these cells in the placental bed, paracrine-mediated inhibition of EVT invasion may also be a factor. Indeed, Hirohata et al. recently demonstrated that soluble factors secreted by *P. gingivalis* inhibit migration of HTR8/SvNeo through Matrigel [96]. Factors responsible for these effects may be components of outer membrane vesicles such as gingipains and fimbriae proteins [97]. This is yet to be determined either *in vitro* or *in vivo*.

## *P. gingivalis* strain-specific effects on APO

It is already well established that *P. gingivalis* strains exhibit different degrees of pathogenicity based on their ability to invade host cells, disseminate to various organ/tissue sites, and induce disease [77,98–100]. This phenomenon also appears to occur in APOs, as indicated by experimental infection in pregnant rats [48]. To date, the two *P. gingivalis* strains that have been used extensively in pregnancy outcome studies are W83 and A7436. Although *in silico* comparison of the whole genome of these strains shows that they are closely related [99], W83 and A7436 differ in the degree and/or type of APO they produce.

*P. gingivalis* strain A7436 has been examined in various species including hamsters, mice, rats, and rabbits [48,56,57,101–103]. The most consistent pathologic feature observed with A7436 infection in pregnant animals is FGR and/or fetal resorption in mice and hamsters [56,57,101,102]. In addition, A7436-induced pathology is primarily restricted to the maternal side of the intrauterine compartment and consists of uterine vasculitis, endometritis, and DDP [48] (Figure 3). Inflammation of fetal membranes (i.e. extensive leukocyte infiltrates into the chorioamnion and/or umbilical cord) is not a common feature of A7436 infection [48]. Interestingly, extremely high doses (10^9^ CFU) of A7436 given intravenously to pregnant rats induces moderate decidual necrosis without any evidence of inflammation in the labyrinth, chorioamnion, or amniotic cavity [48]. Taken together, this suggests that A7436, by itself, is unlikely to trigger the overt inflammatory responses that initiate spontaneous preterm labor [15]. Instead, the pathologic features observed with A7436 are more in line with implantation failure or poor placentation, which often occur with recurrent miscarriage, fetal growth restriction, or preeclampsia [14,75,104,105].

Unlike A7436, W83 is more likely to induce spontaneous preterm delivery, at least in mice [106,107]. In this model, periodontal disease is initiated before breeding by inoculating W83 into molar pulp chambers, which produces increased maternal serum levels of TNF-α, IL-17, IL-6, and IL-1β and spontaneous preterm delivery [106]. Intrauterine lesions associated with this outcome consist of necrosis of the decidua, chorionic plate (fetal side of the placenta), and fetal membranes with moderate infiltration of neutrophils and macrophages into the tissue [106]. Subsequent studies with this animal model showed that fetal membranes expressed higher levels of TNF-α and IL-1β than uninfected controls [107], which would be consistent with spontaneous preterm birth initiated by chorioamnionitis [15]. Although less frequent, FGR is also observed in rodents experimentally infected with W83 [106,108], suggesting that some APOs may be common among most *P. gingivalis* strains.

## Conclusion

Given the effect of *P. gingivalis* strain diversity on APO, determining simply whether or not an individual is colonized is insufficient for establishing who is at risk of having a complicated pregnancy. Furthermore, studies that specifically address the role of *P. gingivalis* virulence mechanisms at the maternal-fetal interface are needed in order to define how this microbe contributes to a wide array of APOs. Regardless of microbial strain diversity, the ability of *P. gingivalis* to disrupt first trimester processes, namely the physiologic remodeling of the uterine spiral arteries, suggests that timing of oral therapies during pregnancy is important and should begin as early as possible including pregnancy or, if possible, prior to conception. Although it has been established that *P. gingivalis* can induce dysbiosis of the oral mucosal microbiota, the impact of this effect on systemic illness, particularly APOs, requires further investigation. The notion that *P. gingivalis* invasion of the maternal-fetal interface could also promote placental dysbiosis linked to APOs is intriguing, but is yet to be tested. Additional studies are warranted to test this hypothesis and to elucidate the microbial mechanisms through which placental dysbiosis occurs.
